# Splice Variants of the RTK Family: Their Role in Tumour Progression and Response to Targeted Therapy

**DOI:** 10.3390/ijms18020383

**Published:** 2017-02-11

**Authors:** Cherine Abou-Fayçal, Anne-Sophie Hatat, Sylvie Gazzeri, Beatrice Eymin

**Affiliations:** Team RNA Splicing, Cell Signaling and Response to Therapies, Institute for Advanced Biosciences, INSERM U1209, CNRS UMR5309, University Grenoble Alpes, Grenoble 38702, France; cherineaf@gmail.com (C.A.-F.); anne-sophie.hatat@univ-grenoble-alpes.fr (A.-S.H.); sylvie.gazzeri@univ-grenoble-alpes.fr (S.G.)

**Keywords:** alternative splicing, angiogenesis, cancer, EGFR, receptor tyrosine kinases, tumourigenesis, targeted therapies, VEGFR

## Abstract

Receptor tyrosine kinases (RTKs) belong to a family of transmembrane receptors that display tyrosine kinase activity and trigger the activation of downstream signalling pathways mainly involved in cell proliferation and survival. RTK amplification or somatic mutations leading to their constitutive activation and oncogenic properties have been reported in various tumour types. Numerous RTK-targeted therapies have been developed to counteract this hyperactivation. Alternative splicing of pre-mRNA has recently emerged as an important contributor to cancer development and tumour maintenance. Interestingly, RTKs are alternatively spliced. However, the biological functions of RTK splice variants, as well as the upstream signals that control their expression in tumours, remain to be understood. More importantly, it remains to be determined whether, and how, these splicing events may affect the response of tumour cells to RTK-targeted therapies, and inversely, whether these therapies may impact these splicing events. In this review, we will discuss the role of alternative splicing of RTKs in tumour progression and response to therapies, with a special focus on two major RTKs that control proliferation, survival, and angiogenesis, namely, epidermal growth factor receptor (EGFR) and vascular endothelial growth factor receptor-1 (VEGFR1).

## 1. Introduction

Growth factors and their receptors are the core components of signal transduction pathways. The majority of growth factor receptors contain extracellular, transmembrane, and cytoplasmic tyrosine kinase domains and transmit the activation signal across the plasma membrane. Binding of growth factors to the extracellular domain of their cognate receptors activates the cytoplasmic tyrosine kinase. Receptor tyrosine kinase (RTK) activation initiates a network of signalling pathways that relay information to the nucleus and other intracellular compartments [1,2]. RTKs are key regulators of critical cellular processes, such as proliferation and differentiation, cell survival and metabolism, organ morphogenesis, neovascularization, cell migration, and tissue repair and regeneration [3,4]. In normal cells, RTK activity is strictly regulated. Numerous diseases result from genetic changes or abnormalities that alter the activity, abundance, cellular distribution, or regulation of RTKs. Cancers frequently display deregulation or constitutive activation of RTKs, and abnormal RTK signalling is an important feature of tumour initiation and progression. Therefore, RTKs appear as promising molecular targets in cancer.

It is now well accepted that approximately 90% of human multi-exon genes are regulated by pre-mRNA alternative splicing [5,6]. In addition, an increasing number of studies have shown that misregulation of alternative splicing occurs in many pathologies, such as cancers ([7] for review). Target genes whose splicing is deregulated in tumours include those encoding various RTKs and their ligands (Table 1).

Alternative splicing of RTKs has several biological consequences. First, it can modify the subcellular distribution of RTKs, thereby affecting their activity. As an example, Vorlova and colleagues identified 31 decoy receptors produced by intron retention and activation of intronic poly(A) sites in 16/20 RTK family members [27]. These truncated RTKs are devoid of their transmembrane and intra-cytoplasmic tyrosine kinase domains. Therefore, they are considered dominant-negative receptors that act either by sequestering RTK ligands or by inhibiting other RTKs through heterodimerization [27]. Another example is the alternative splicing of ErbB4, which produces both juxtamembrane (JM-a and JM-b) and cytoplasmic (CYT-1 and CYT-2) isoforms that are differentially endocytosed [16]. Second, alternative splicing can modify the affinity of the RTKs for their ligands, providing a specific signalling for each splice variant. This is the case for the fibroblast growth factor receptor 2 (FGFR2) that is expressed as two different splice isoforms, FGFR2IIIb and FGFR2IIIc, depending on whether exon 8 or exon 9 is skipped [30]. These FGFR2 splice variants do not bind the same FGF ligands. Interestingly, FGFR2 exon switching from the IIIb to the IIIc isoform has been observed during epithelial cell tumour progression, notably in breast cancer [31]. Moreover, expression of FGFR2IIIc was associated with epithelial to mesenchymal transition [32]. Therefore, alternative splicing of RTKs may contribute to cellular reprogramming and the generation of cancer cells with more invasive features. This was also seen with a splice variant of Ron, the tyrosine kinase receptor for the macrophage-stimulating protein. This Ron isoform, named RonΔ65, is generated through the skipping of exon 11, leading to a kinase that is constitutively active in the cytoplasm, even in the absence of Ron ligand. This splicing event is controlled by the splicing factor SRSF1. Importantly, the accumulation of RonΔ65 is associated with a metastatic phenotype in human colorectal and breast carcinomas [33]. Although increasing evidence indicates that alternative splicing of RTKs may have a critical role during tumourigenesis, many questions still remain to be elucidated. For example, less is known about the upstream regulators and extracellular cues that control the splicing of these RTKs. In addition, as alternative splicing may be dependent on tumour types, it remains to be determined whether alternative splicing of RTKs may play distinct roles depending on the tumour context, and which specific signalling networks are activated by these splice variants in this setting. Last but not least, it is largely unknown whether, and how, alternative splicing of RTKs may be involved in the primary and/or acquired resistance of tumour cells to targeted therapies widely used in the clinic. As an example, the splice variant derived from exon 16 skipping of the HER2 receptor, called Δ16HER-2, has been associated with malignant transformation and resistance to trastuzumab, an anti-HER2 monoclonal antibody, in breast cancers [34]. Investigating these questions is crucial, as this could lead to the identification of new prognostic markers and help define new therapeutic strategies. 

This review will focus on two key RTKs, namely, the epidermal growth factor receptor (EGFR) and the vascular endothelial receptor (VEGFR1), as an illustration of how alternative splicing of RTKs can be involved in both tumourigenesis and response to therapies. These RTKs were chosen because they are critical regulators of tumour proliferation, survival, and angiogenesis, and because they are targeted by pharmacological compounds widely used in the clinic to treat cancer patients. 

## 2. Splicing of EGFR: An Alternative Method to Control Tumour Progression

**Figure 1:**
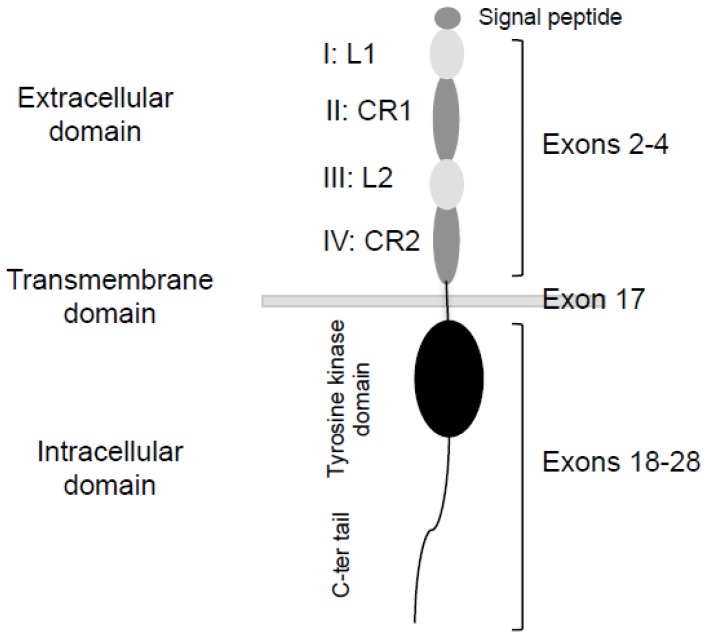
Schematic structure of the EGFR monomer. L: Ligand binding domain. CR: Cysteine-rich domain.

EGFR is a transmembrane protein with tyrosine kinase activity (Figure 1). Its structure includes an extracellular domain with four domains repeated two by two: L1 (I), CR1 (II), L2 (III), and CR2 (IV). The L1 and L2 domains are required for ligand fixation, and the CR1 and CR2 domains (cysteine-rich) increase the affinity of the ligand for its receptor and allow dimerization with the second receptor of the dimer [35,36]. Ligand binding to the extracellular domain triggers the dimerization of the receptor and induces its autophosphorylation. Dimerization of the receptor induces a cascade of phosphorylation leading to the activation of proliferation and survival pathways. Dysregulated EGFR signalling contributes to the formation of many epithelial malignancies in humans. Therefore, EGFR is an attractive candidate for targeted therapy, as it is often overexpressed on the surface of cancer cells.

### 2.1. Soluble EGFR Variants

**Figure 2:**
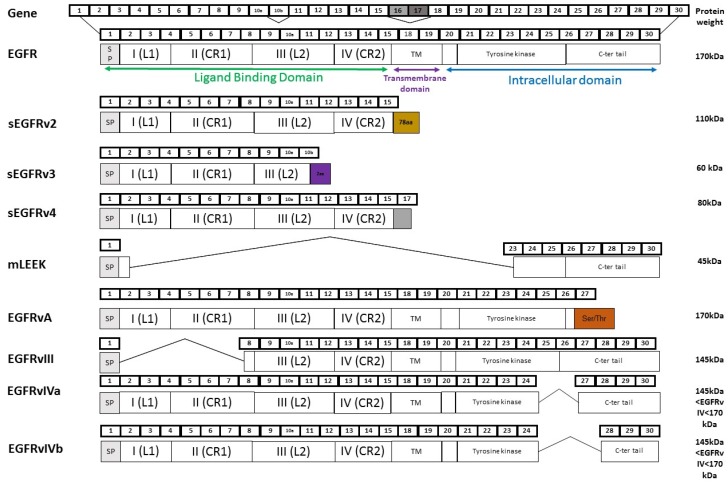
EGFR and its splicing variants. Alternative splicing of EGFR generates eight variants including those that encode soluble isoforms, sEGFRv2, sEGFRv3, and sEGFRv4, and those can encode non-soluble isoforms, mLEEK, EGFRvA, EGFRvIII, EGFRvIVa, and EGFRvIVb. For each splice variant, the number of exons (upper) and functional domains of the protein (lower) are represented. L: ligand binding, CR: Cysteine-Rich.

*EGFR* contains 30 exons and generates different mRNAs (Figure 2). Variant 1 mRNA is devoid of exons 16–17 and encodes the 170-kDa full-length EGFR. In addition to full length receptor, normal and tumour cells express soluble EGFR isoforms (sEGFR) that contain only the extracellular domain of EGFR. These sEGFR proteins can result either from alternative splicing or from proteolytic cleavage of the receptor [37,38]. Alternative splicing of the EGFR gene generates three variants that encode 110-kDa, 80-kDa, and 60-kDa sEGFR isoforms [39,40,41]. Soluble EGFR isoforms have been described in normal tissues, but they are highly expressed in human placenta; the 110-kDa isoform is the major one, suggesting a potential role in this tissue. Intra-tumoral and/or plasma/serum levels of sEGFR have been examined in tumour patients, but in most studies the data reflect overall sEGFR. Baron and colleagues were the first to report the presence of sEGFR in epidermal ovarian cancer (EOC) patients. They observed that the level of sEGFR is lower in patients harbouring EOC compared to healthy patients [42]. Similar results were reported in breast tumours [43] and non-small cell lung cancer (NSCLC) patients [44], suggesting that expression of sEGFR may have a physiological and protective role against cancer development. In this respect, sEGFR was proposed as a potential negative biomarker for the early detection in NSCLC [45]. On the other hand, other studies found significantly elevated sEGFR levels in cervical and gastric carcinoma compared to the healthy population [46], suggesting that sEGFR could also be a positive tumour marker. Moreover, clinical data regarding the potential of sEGFR as a prognostic biomarker in patients with various solid tumours remain controversial. Lower sEGFR levels were associated with reduced survival in advanced NSCLC patients [47] and in meningioma [12]. In agreement with an antiproliferative function, sEGFRs were reported to inhibit tumour cell proliferation and migration in NSCLC cell lines [48]. On the other hand, high levels of sEGFR were associated with a shorter survival rate in gastric cancer [46]. Therapeutic predictive values of sEGFR levels in blood have also been shown in some cancers. High levels of sEGFR may predict the response to tyrosine kinase inhibitors in colorectal cancer [49]. Low sEGFR levels are associated with shorter overall survival in patients with metastatic breast cancer treated with chemotherapy [50]. As a whole, these results highlight sEGFR as an interesting biomarker for diagnosis, prognosis, and response to therapy, but its overall role in cancer is still to be defined. Further investigations aiming at deciphering the identity (110-kDa, 80-kDa and/or 60-kDa EGFR isoforms) and origin (alternative splicing and/or proteolytic cleavage of EGFR) of the sEGFR isoforms expressed in these biological samples will probably help to understand the clinical data.

### 2.2. EGFRvIII

EGFRvIII is the most common EGFR splice variant. It skips exons 2–7 and encodes a protein of 145 kDa that is devoid of the extracellular ligand-binding domain. As a consequence, EGFRvIII is unable to bind the soluble ligands of EGFR. Nevertheless, EGFRvIII shows constitutive tyrosine phosphorylation through basal dimerization, activates multiple downstream signalling pathways [51] and exhibits a high tumourigenic potential [52]. It is often co-expressed with wild-type EGFR, especially in tumours with EGFR amplification [53], and almost all published studies report the absence of EGFRvIII expression in normal tissues [54]. EGFRvIII has been mainly studied in high-grade brain tumours such as glioblastoma multiforme (GBM), in which EGFRvIII is detected at an overall frequency of 25%–64% [13]. EGFRvIII expression has also been reported in human tumours outside the central nervous system, including lung, breast and HNSCC [13], but its frequency and significance remain controversial. Some studies have identified EGFRvIII as a marker of poor prognosis in GBM patients [55], but others did not find an association between EGFRvIII and patients outcomes [56]. EGFRvIII expression has been implicated in the progression of breast cancer and could play a role in tumour metastasis [57]. EGFRvIII signalling has been reported to enhance the tumourigenicity of GBM, breast, lung and ovarian tumour cells, among others. The pro-tumourigenic effects of EGFRvIII seem to be mediated by pathways that are activated by wild-type EGFR, including RAS/RAF/mitogen-activated protein kinase (MAPK), phosphatidylinositol3-kinase (PI3K)/AKT, signal transducer and activator of transcription 3 (STAT3), and nuclear factor κB (NF-κB) [13], but there is emerging evidence that EGFRvIII can co-activate other cell-surface receptors [58]. Therefore, EGFRvIII signalling plays a role in tumourigenesis and tumour progression by mediating cell survival, proliferation, motility and invasion. Interestingly, a recent study also showed that EGFRvIII displays cancer stem cell-specific expression and can be used to specifically target this population [59].

The therapeutic potential of targeting EGFRvIII is becoming apparent, especially in brain tumours. A number of therapeutic approaches, some of them being commonly used to also target wild-type EGFR, have shown pre-clinical and/or clinical promise. Tyrosine kinase inhibitors showed good pre-clinical results with inhibition of tumour growth, angiogenesis, survival, and proliferation [60]. Response rates in glioblastoma patients were disappointing for many inhibitors [61]. Antibody-based therapies demonstrated efficacy in vitro, but their systemic injection failed to block tumour growth in vivo [62]. Various clinical trials that use conjugated antibodies with toxins or radioactive isotopes gave positive results with improvement of median survival [63]. Pre-clinical trials of RNA therapies based on the use of antisense oligonucleotide, RNA interference and ribozyme also yielded encouraging in vitro and in vivo results [64]. However, intra-tumoral heterogeneity of EGFR expression, development of resistance mechanisms by the tumour cells and low efficiency of therapeutic drugs to bypass the blood-brain barrier have limited the clinical utility of these therapies [61]. Immune therapy using vaccines is a promising treatment. Several laboratories have shown that a peptide vaccine targeting the EGFRvIII antigen can effectively reduce tumour progression in pre-clinical models [65]. Furthermore, several phase II clinical trials demonstrated improved survival and specific immune response to EGFRvIII in patients treated with the vaccine [66]. Clinical trials testing the efficiency of vaccine combined with EGFR-targeted therapy in brain tumour patients are underway. The role of anti-EGFRvIII therapy in other tumour types is still to be addressed.

### 2.3. EGFRvIV

The carboxyl terminal deletion mutants collectively called EGFRvIV lack either exons 25–27 (EGFRvIVa) or exons 25–26 (EGFRvIVb) [67]. The deletion initiates immediately downstream of the kinase domain. Little is known about the oncogenic potential of these EGFR mutants. It has been shown that internal deletions of EGFRvIV enhance basal kinase activity and confer tumourigenic growth in animals [68]. Furthermore, like EGFRvIII, EGFRvIV mutants display basal dimerization, enhanced basal kinase activity and increased stability due to association with HSP90. Constitutive downstream signalling by the EGFRvIV mutants includes activation of STAT3, MAPK, and AKT pathways, but it is suggested that the mutants activate different cellular programs and that distinct pathways are recruited by EGFRvIII and the EGFRvIV mutants [68]. The EGFRvIV mutants have been identified in glioblastoma multiforme, and to date there is no evidence of their expression in other cancer types.

### 2.4. EGFRvA

Structurally, EGFRvA is characterized by the substitution of exon 28 of EGFR with a serine/threonine-rich sequence [69]. Compared to wild-type EGFR, EGFRvA is more stable because of its decreased binding to c-Cbl [70], and it promotes cancer migration and invasion more significantly through activation of the STAT3 pathway and autocrine production of heparin-binding EGF [69]. EGFRvA is highly expressed in placenta and only slightly expressed in other normal tissues. Many cancer cell lines and tissues have EGFRvA, suggesting a role in tumour development. The detection of EGFRvA is positively correlated with tumour grade and with adverse prognosis in glioma patients, more significantly than EGFR [69]. Thus, EGFRvA plays a critical role in the tumour progression of gliomas and might be a good therapeutic target for cancer treatment.

### 2.5. mLEEK

Recently, a new alternative splicing variant called mLEEK was identified [15]. This protein lacks the extra-cytoplasmic, transmembrane and ATP binding site of the tyrosine kinase domain of EGFR. mLEEK is widely expressed in normal tissues and is overexpressed in human tumours, including those from ovary, skin, and lung. Interestingly, this variant localizes in the nucleus and co-regulates target gene expression that controls the unfolded protein response (UPR). Thus, mLEEK is able to favour cell growth in unfavourable conditions, making it an interesting target for future therapeutic development.

## 3. Alternative Splicing of VEGFR1: From Anti-Angiogenic to Pro-Angiogenic Factors

The VEGF (vascular endothelial growth factor) family is composed of seven glycoproteins: VEGF-A, VEGF-B, VEGF-C, VEGF-D, VEGF-E, VEGF-F, and PlGF (placental growth factor), which exert different biological functions, such as angiogenesis and lymphangiogenesis, by binding to three VEGF receptors (VEGFRs): VEGFR1, VEGFR2, and VEGFR3 [71]. It has been shown that the entire family of VEGF ligands and VEGFRs display alternative splice variants. For instance, alternative splicing of VEGF-A generates multiple splice variants known as VEGFxxx (where xxx is the total number of amino acids in the mature protein) (see [72,73] for reviews). At the functional level, these VEGFxxx splice variants differentially bind heparin-containing proteoglycans that are present at the cell surface or in the extracellular matrix. As a consequence, VEGFxxx splice variants are more or less diffusible [74]. In addition, they display different binding affinities for their VEGFR1/VEGFR2 receptors and their co-receptors, neuropilins, thereby being more or less potent activators of VEGFR signalling pathways [75]. Interestingly, in 2002, a new family of VEGF-A splice variants, termed VEGFxxxb, was discovered [76]. VEGFxxxb isoforms result from the selection of a distal splice site in the last exon of VEGF-A and the inclusion of a new exon called exon 8b. At the protein level, VEGFxxxb share 94%–98% homology with VEGFxxx and differ only at the level of six amino acids in the C-terminal end. VEGFxxxb can bind VEGFR receptors with the same affinity than VEGFxxx, but they are unable to bind neuropilins and to fully activate VEGFR-dependent signalling pathways [77,78]. As a consequence, while VEGFxxx splice variants are angiogenic factors overexpressed in many tumours [74], VEGFxxxb appear to act as anti-angiogenic factors competing with and inhibiting all the effects (proliferation, migration, survival) of VEGFxxx on endothelial cells. In addition to VEGF-A, VEGFRs are also subjected to alternative splicing. Here, we will discuss only the role of the VEGFR1 splice variants, but it is interesting to note that a soluble VEGFR2 splice variant has also been identified and shown to act as an inhibitor of lymphatic vessel growth [79].

### 3.1. VEGFR1 Splice Variants

**Figure 3:**
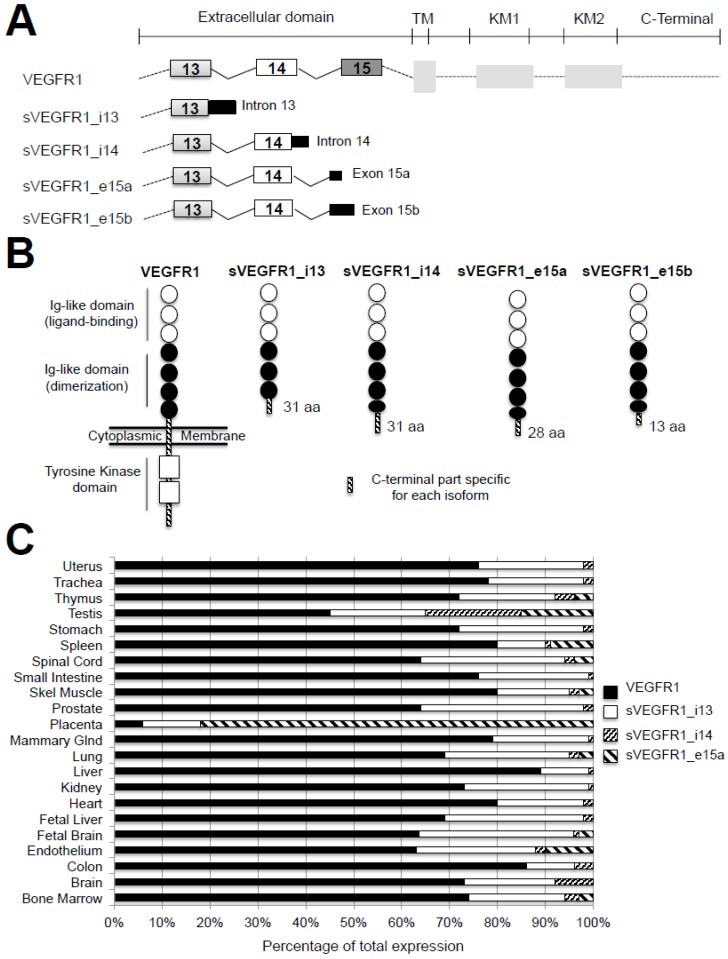
The different VEGFR1 splice variants, proteins and expression in tissues. (**A**) Schematic representation of full-length VEGFR1, sVEGFR1_i13, sVEGFR1_i14, sVEGFR1-e15a, and sVEGFR1-e15b mRNAs. Exons and introns are shown. TM: Transmembrane domain, KM1: ATP-binding domain, KM2: phosphotransferase domain; (**B**) Schematic representation of full-length VEGFR1 and sVEGFR1s proteins. Each splice variant isoform contains the first six extracellular Ig-like domains of VEGFR1, with (sVEGFR1-i14, sVEGFR1-e15a, sVEGFR1-e15b) or without (sVEGFR1-i13) a part of the last Ig-like domain, followed by a specific C-terminal end represented as a hatched box in the figure (adapted from [82]). aa represents the number of amino acids contained in the specific C-terminal part; (**C**) Percentage of expression of VEGFR1, sVEGFR1-i13, sVEGFR1-i14, and sVEGFR1-e15a mRNAs as indicated, according to the tissue type. sVEGFR1-e15b mRNA is undetectable in most of these tissues (adapted from [84]).

To date, four alternative splice variants of VEGFR1, named sVEGFR1-i13, sVEGFR1-i14, sVEGFR1-e15a, and sVEGFR1-e15b, have been described [26] (Figure 3A). These variants have the same transcription start site as *Vegfr1*. However, while *Vegfr1* mRNA contains 30 exons, *sVegfr1* mRNAs share only the first 13–15 exons, depending on the variant, and encode truncated proteins that are devoid of their transmembrane and intra-cytoplasmic tyrosine kinase domains. This is the reason why they are considered soluble decoy receptors and annotated as sVEGFR1. The sVEGFR-1_v1 or sVEGFR1-i13 was initially discovered in 1993 as a 100-kDa protein highly expressed in endothelium [26]. It results from an inclusion of intron 13 followed by a premature polyadenylation. This splice variant is the shortest and the most studied among sVEGFR1s. The sVEGFR-1_v2 or sVEGFR1-e15a contains the first 14 exons and a new terminal exon (denoted exon 15a) derived from an intronic sequence [80], while the sVEGFR-1_v3 or sVEGFR1-e15b contains a new 3′-end denoted exon 15b distinct from the variant v2 [81]. The sVEGFR-1_v4 or sVEGFR-1-i14 results from skipping a splice site, leading to the extension of exon 14 followed by a polyadenylation [81].

At the protein level, sVEGFR1-i13 protein contains the first six Ig-like domains of the full-length VEGFR1 that correspond to 657 amino acids. Therefore, it possesses the ligand binding domain but lacks the seventh Ig-like domain, as well as the transmembrane and the tyrosine kinase regions of VEGFR1 (Figure 3B). In contrast, it has a unique C-terminal sequence composed of 31 amino acids. This specific sequence is highly conserved in mammals and differs only at the level of two amino acids between mouse and human [82]. The molecular weight of sVEGFR1-i13 varies depending on the cell type, being 110 kDa in HUVEC (human umbilical vein endothelial cells) and HDMEC (human dermal microvascular endothelial cells) and 120–130 kDa in COLO-800 melanoma cells [83]. These differences might reflect various glycosylation levels. For sVEGFR1-i14, sVEGFR1-e15a, and sVEGFR1-e15b, the extension of the open reading frame is low (93, 84, and 33 bp, respectively), and the protein is modified after amino acid 706, leading to a truncated protein between the two last Ig-like domains. These variants contain specific C-terminal extremities of 31, 28, and 13 amino acids, respectively [82] (Figure 3B).

### 3.2. Expression and Regulation of sVEGFR1 in Tissues

The sVEGFR1 isoforms are secreted by several cell types, including endothelial cells [85], smooth muscle cells [86], monocytes and macrophages [85], trophoblasts of the placenta [85], and proximal tubular cells of the renal epithelium [87], as well as by various cancer cells [88]. Each cell type expresses different levels of sVEGFR1 variants. For example, smooth muscle cells predominantly express sVEGFR1-i14, while endothelial cells mainly express sVEGFR1-i13 [86]. sVEGFR1s are also differentially expressed according to the organs (Figure 3C). For example, the level of sVEGFR1-i13 is forty times greater in the placenta than in the heart, kidneys, and lungs. In addition, the sVEGFR1-e15a is the most abundant isoform in the placenta [83]. Taken together, these data suggest that the role of each sVEGFR1 might be highly dependent on the tissue and/or the cell type. Although extracellular signals that control sVEGFR1 expression are largely unknown, endogenous stimuli, such as growth factors [89,90], cytokines [91], hypoxia [92], and miRNAs [93], have been shown to induce the expression of sVEGFR1-i13. Moreover, a cooperative role between the arginine demethylase and lysine hydroxylase JMJD6 (JuMonJi Domain containing-protein 6) and the splicing factor U2AF65 [94], as well as inhibition of the NOTCH1 signalling pathway [95], have been reported to up-regulate the expression of sVEGFR1-i13. These studies have been performed in endothelial cells, dendritic cells, macrophages, cytotrophoblasts, placenta, or retina. To date, the upstream signals that control sVEGFR1 expression in cancer cells remain unknown.

### 3.3. Role of Vascular Functions of sVEGFR1s in Physiological and Pathological Conditions

It was first shown that sVEGFR1s, which are devoid of tyrosine kinase domains (Figure 3B), exert anti-angiogenic functions through inhibition of VEGF-A/VEGFR signalling. Two mechanisms of action were proposed: the sequestration of VEGF-A ligand and/or its heterodimerization with VEGFR2 receptor [96,97]. Based on its vascular effects, both physiological and pathological functions have been attributed to sVEGFR1. Physiologically, the non-vascularization of the cornea supports optical clarity. Among many anti-angiogenic molecules (angiostatin, endostatin, etc.), sVEGFR1 is the only one required to inhibit the pro-angiogenic effects of VEGF-A in the cornea [98,99]. During normal pregnancy, it was also proposed that sVEGFR1 maintains the vascular integrity of the placenta by sequestering the excess of VEGF-A [100]. In addition, sVEGFR1 has an anti-oedema role through its ability to interfere with the vascular permeability function of VEGF-A [101,102]. Last but not least, sVEGFR1 displays protective anti-inflammatory functions because it prevents the activation and migration of monocytes and macrophages [103]. Conversely, sVEGFR1 has also been implicated in many vascular pathologies. The most described is preeclampsia, a pregnancy-specific disorder characterized by hypertension and proteinuria occurring in the second half of pregnancy and resulting in neonatal or maternal morbidity and mortality. It has been shown that plasma levels of both sVEGFR1-i13 and sVEGFR1-e15a proteins increase five weeks before the onset of preeclampsia symptoms [104]. However, the sVEGFR1-e15a variant appears to be mainly involved in this pathology [82,86]. In preeclampsia, various studies have shown that hypoxia, oxidative stress, and an excess of VEGF-A in the endometrium regulate the production of sVEGFR1 [100,104,105]. Moreover, increased expression of sVEGFR1 was observed in patients with wound healing defects [106], as well as in patients with idiopathic pulmonary arterial hypertension [107] or adult respiratory distress syndrome [108]. Both pathologies are associated with abnormal vascular permeability.

### 3.4. Role of sVEGFR1 in Tumour Progression

Tumour neo-angiogenesis is a prerequisite for tumour progression in most solid cancers. In different xenografted tumours in mice (melanoma, lung cancer, fibrosarcoma, glioblastoma), exogenous administration of sVEGFR1 through different approaches (transfection, adenovirus infection, or use of recombinant protein) was found to inhibit tumour growth and neo-angiogenesis and to increase the survival rate [109,110,111,112]. However, contrasting results were also reported. Expression of sVEGFR1 was able to rescue the aberrant morphogenesis of embryonic vessels that occurs in VEGFR1 knock-out mice through stimulation of vascular sprouting and endothelial cell migration [113]. In addition, sVEGFR1-i13 was shown to induce the adhesion and migration of endothelial cells through interaction with α5β1 integrin and activation of rac1 and radixin, a substrate of PKC [114]. Taken together, these data extended the previous view regarding the anti-angiogenic functions of sVEGFR1 by providing evidence that sVEGFR1 can act as both a positive and a negative regulator of the angiogenic process.

Other studies demonstrated that sVEGFR1 can also act on tumour cells themselves. sVEGFR1 was recently reported to induce non-apoptotic death in ovarian or colorectal cancer cell lines and to promote tumour regression in an ovarian carcinoma mouse model [115]. Conversely, Ruffini and collaborators showed that sVEGFR1-i13 is produced in the extracellular matrix of melanoma cells and induces cell adhesion by activating the VEGF-A/VEGFR2 signalling pathway [116]. In addition, involvement of sVEGFR1 in metastatic processes was highlighted. Indeed, high levels of VEGF-A and sVEGFR1 were reported in metastatic breast cancer compared to non-metastatic cancers [117]. In melanomas, high levels of sVEGFR1-i13 are depicted with respect to human melanocytes, but there is a lower level of sVEGFR1i-13 in skin metastases compared to primary tumours [116]. 

### 3.5. sVEGFR1 as a Prognostic Biomarker in Cancer

Several studies have analysed intra-tumoral and/or plasma/serum levels of sVEGFR1 in tumours. However, few of them have examined which sVEGFR1 splice variant is expressed, and most of the data reflect overall sVEGFR1. It has been shown that sVEGFR1 protein is overexpressed in many types of cancer, including glioblastoma, leukaemia, melanoma, colorectal, breast, renal, hepatocellular, head and neck, and lung carcinoma [88,116,118,119,120,121,122,123,124]. In this setting, high levels of sVEGFR1 are often correlated with poor prognosis. In addition, the levels of both sVEGFR1 and VEGF-A have been previously used to determine the prognosis of cancer patients, but the results remain controversial. As an example, in lung cancer patients, high levels of both sVEGFR1 and VEGF-A are correlated with poor prognosis and with very advanced stages [124]. In glioma, leukaemia, breast, or pancreatic cancer, high levels of VEGF-A combined with low levels of sVEGFR1 in the serum, plasma or tumour extracts are correlated with high grade, reduced survival rate and/or poorer response to therapy [118,125,126,127]. In contrast, Toi et al. reported that when the level of sVEGFR1 is 10 times higher than that of VEGF-A, this correlates with a better prognosis in breast cancer [120]. Therefore, the balance between sVEGFR1 and VEGF-A levels appear to be important for clinical outcome.

### 3.6. sVEGFR1 as a Biomarker of Tumour Response to Therapies

sVEGFR1 has also been investigated for its potential as a determinant of response to anti-angiogenic therapies, and many studies have quantified its plasma and/or serum levels before and/or after treatment [128]. Interestingly, high basal plasma levels of sVEGFR1 were often inversely correlated with response to bevacizumab or VEGFR-TKI in colorectal cancer (vandetanib plus cetuximab/irinotecan), hepatocellular carcinoma (cediranib), sarcoma (sorafenib), lung or renal cancer (bevacizumab), or triple-negative breast cancer [28,121,129,130]. In breast cancers, the resistance to bevacizumab was directly correlated with pericyte coverage, a marker of vascular normalization, thereby suggesting that the vascular functions of sVEGFR1 may account for its negative impact on tumour response to anti-angiogenic therapies [129]. However, and making things slightly more complicated, it was also shown that sVEGFR1 expression level either decreased or increased upon treatment in triple-negative breast, rectal, or metastatic colorectal cancers [28,129,131,132]. These data indicated that these therapies regulate sVEGFR1 expression level in opposite ways depending on the tumour type. In addition, combination of sVEGFR1 levels with those of other angiogenic regulators has been used to predict response. As an example, in patients with advanced colorectal cancer treated with bevacizumab and chemoradiation, high levels of sVEGFR1 and low levels of VEGF-A correlated with abnormal vascularity and poor response despite fewer side effects [133]. In advanced non-squamous cell lung carcinoma, high levels of PlGF and sVEGFR1 before treatment were associated with a poor response. After treatment, although the level of PIGF remained high, the level of sVEGFR1 transiently decreased [29]. Overall, these data highlight the potential role of sVEGFR1 as a biomarker of resistance to anti-angiogenic therapies, mainly through vascular effects [131,134,135]. As sVEGFR1 also acts on tumour cells themselves, it remains to be determined whether this autocrine function also contributes to the resistant phenotype.

Finally, and based on the anti-angiogenic functions of sVEGFR1, different therapeutic strategies have been elaborated in tumours to design “sVEGFR1-like” therapies, among them the VEGF-Grab. This soluble receptor comprises the second and third Ig-like domains of VEGFR-1 that are combined to the Fc fragment. The Ig-like domain 3 of VEGFR1 is glycosylated because the positive charges induce non-specific binding to the extracellular matrix. Interestingly, VEGF-Grab demonstrated anti-angiogenic, anti-tumour and anti-metastatic effects, suggesting that its clinical use could result in promising anti-angiogenic effects [136]. In addition, it was shown that morpholino constructs targeting the *Vegfr1* mRNA exon13/intron13 junction promote the production of sVEGFR1 over membrane-bound VEGFR1 and decrease tumour neovascularization in vivo [137]. Manipulating sVEGFR1 expression could offer translational potential for therapy, although it is important to keep in mind that overexpressing sVEGFR1 could also trigger deleterious effects, depending on the context and/or the tumour type.

## 4. Conclusions

Enhanced RTK signalling is a driving force in many human malignancies. Although much work has been completed concerning the identification and functional consequences of RTK mutations or copy number variations in cancer, less is known about the role of RTK splice variants. Management of tumour patients with primary/acquired resistance to targeted therapies remains a significant challenge with therapeutic, social, and economic impacts. Thorough understanding of the biological significance of alternative splicing of RTKs and components of RTK signalling pathways could allow for the identification of new prognosis biomarkers, as well as the definition of alternative therapeutic strategies. Drugs targeting the spliceosome machinery are currently being tested in pre-clinical trials and have already demonstrated anti-tumour efficacy. As an example, spliceostatin A, or its analogue meayamycin B (MAMB), slows down the growth of vemurafenib-resistant tumours by decreasing the amount of the resistant BRAF3-9 splice variant [138,139]. In addition, treatment with the spliceosome inhibitor E7107 results in substantial reductions in leukaemic burden, specifically in patient-derived xenograft AMLs carrying mutations of spliceosomal components [140]. These results provide a rationale for targeting RNA splicing in cancer in combination or not with RTK-targeted therapies.

## Figures and Tables

**Table 1:** Examples of pre-mRNA alternative splicing (AS) of various receptor tyrosine kinases and functional consequences.

RTK	Splicing Events	Functional Consequences	References
**ALK**	Skipping of exons 2–3 Skipping of exons 4–11	Truncated proteins with increased constitutive kinase activity and transformation potential in neuroblastoma	[8]
	Skipping of exon 23 or exon 27	Truncated proteins lacking the full kinase domain of ALK in Non Small Cell Lung Carcinoma	[9]
**AXL**	Skipping of exon 10	Shorter AXL protein with same transforming potential as full-length AXL	[10]
**DDR**	Exon skipping or inclusion	Distinct binding partners Differential activation by collagen	[11]
**EGFR**	Inclusion of exon 10, 9a, 16 or 17	Soluble receptors acting as negative regulators of EGFR signalling	[12]
	Skipping of exons 2–7	Constitutively active receptor	[13]
		Enhanced signalling, survival, and tumourigenicity	[14]
	Skipping of exons 2–22	Enhanced migration and invasion Cancer stem cells marker	[15]
**ERB4**	N- and C-terminal alternative splicing generating four isoforms	Modulation of sub-cellular localization and partner binding	[16]
**FGFR**	Mutually exclusive exon 8 or 9	Generation of distinct extracellular Ig-like domain III with distinct affinity for FGF ligands	[17]
		Induction of Epithelial to Mesenchymal Transition (EMT), invasion and motility	[18]
**INSR**	Skipping or inclusion of exon 11	Generation of INSR-A and INSR-B splice variants that respond differentially to IGF-II and insulin ligands and differentially activate the RAS/MAPK pathway	[19]
**MET**	Skipping of exon 14	Activation of MET kinase activity Oncogenic transformation	[20]
		Increased sensitivity to MET inhibitors	[21]
**RET**	3′-end alternative splicing generating multiple isoforms that differ in their C-terminal domain	Modulation of signalling partner binding Distinct sub-cellular localization and trafficking properties Transforming capacity	[22]
**RON**	Skipping of exon 11	Constitutively active receptor Enhanced signalling, invasion, motility	[23]
	Skipping of exons 15–19, 16–19, 16–17 and 16	Truncated protein lacking active kinase domain Dominant negative isoforms in lung cancers.	[24]
**NTRK**	Skipping of exons 6, 7 and 9	Constitutively active receptor Oncogenic function in neuroblastoma	[25]
**VEGFR**	Intron retention followed by premature polyadenylation	Soluble decoy receptor acting as negative regulator of VEGFR signalling	[26,27]
		Increased resistance to anti-angiogenic therapies	[28,29]

ALK: Anaplastic Lymphoma Kinase; DDR: Discoidin Domain Receptor; FGFR: Fibroblast Growth Factor Receptor; INSR: Insulin Receptor; RON: Receptor d’Origine Nantaise; NTRK: Neurotrophic Tyrosine Kinase Receptor.
